# A rare combination of disease presentation of HIV positive person, attending Government Hospital, Chennai – a case report

**DOI:** 10.1186/1471-2334-12-S1-P37

**Published:** 2012-05-04

**Authors:** Jasmine S Sundar, T Uma, S Parameswari, S Sekar, N Kabilan, Mayilvahanan Natarajan

**Affiliations:** 1 Department of Epidemiology, The TN Dr. MGR Medical University, Chennai, Tamil Nadu, India; 2 Tamilnadu State AIDS Control Society, Chennai, Tamil Nadu, India

## Background

Liver disease in the HAART era is one of the leading causes of morbidity and mortality in HIV-infected individuals. Non cirrhotic portal hypertension is an uncommon condition, although increasingly being reported in HIV-infected along with gastrointestinal bleeding. This is a rare case of pre ART patient.

## Case history

A 36 year male with complaints of breathlessness, abdominal distension, intermittent fever, pedal edema and bleeding per rectum for 10 days. He was chronic smoker, alcoholic, extramarital exposure and has occupational history of tar. Recently diagnosed for HIV and ART naïve patient. Spouse and their children’s HIV status were not known.

## Examination

General examination showed emaciated, oriented, febrile, pallor, icteric, tenderness and swelling present in right thigh. Impetigo scars seen over the both lower limbs. Clubbing and bilateral pitting pedal edema was present. Oral examination shows fissures in tongue. Systemic examination reveals cardio vascular, central nervous system was normal. Respiratory system shows occasional crepts. Hepatosplenomegaly was present with ascitis.

## Investigations

Hematology, urea, sugar and electrolytes were normal. Liver function tests, prothrombin time, international normalized ratio and 24 hrs protein was moderately elevated. Colour Doppler and duplex examination showed no flow in femoral, popliteal, anterior tibial veins and sluggish flow in postier tibial vein. Chest X ray showed no abnormalities and sputum for AFB was negative. His CD4 count is 115 cells/mm^3^.

## Hospitalization

Patient was admitted in the hospital, and treated symptomatically with antibiotics, anti coagulants and diuretics.

## Diagnosis

HIV, decongestive chronic liver disease, deep vein thrombosis, portal hypertension, jaundice and hemorrhoids.

